# Workplace bullying increases the risk of anxiety through a stress-induced β2-adrenergic receptor mechanism: a multisource study employing an animal model, cell culture experiments and human data

**DOI:** 10.1007/s00420-021-01718-7

**Published:** 2021-06-02

**Authors:** Dhaksshaginy Rajalingam, Ingeborg Nymoen, Henriette Nyberg, Morten Birkeland Nielsen, Ståle Valvatne Einarsen, Johannes Gjerstad

**Affiliations:** 1 Department of Psychosocial Science, University of Bergen, Bergen, Norway; 2 National Institute of Occupational Health, Oslo, Norway

**Keywords:** Social stress, Bullying, Rat, Human, rs1042714, Anxiety

## Abstract

**Objectives:**

Several studies show that severe social stressors, e.g., in the form of exposure to workplace bullying in humans, is associated with negative mental health effects such as depression and anxiety among those targeted. However, the understanding of the underlying biological mechanisms that may explain the relationship between exposure to bullying and such negative health outcomes is scarce. The analyses presented here focus on understanding the role of the β_2_-adrenergic receptors (ADRB2) on this association.

**Methods:**

First, a resident-intruder paradigm was used to investigate changes in circulating norepinephrine (NE) in rat serum induced by repeated social defeat and its relationship with subsequent social behavior. Second, the direct effects of the stress-hormones NE and cortisol, i.e., synthetic dexamethasone (DEX), on the ADRB2 expression (qPCR) and monocyte chemoattractant protein-1 (MCP-1) release (immunoassay) was examined in cultured EL-1 cells. Third, in a probability sample of 1052 Norwegian employees, the 9-item short version of the Negative Acts Questionnaire—Revised (S-NAQ) inventory, Hopkins Symptom Checklist and genotyping (SNP TaqMan assay) were used to examine the association between social stress in the form of workplace bullying and anxiety moderated by the ADRB2 genotype (rs1042714) in humans.

**Results:**

The present study showed a clear association between reduced social interaction and increased level of circulating NE in rats previously exposed to repeated social defeat. Parallel cell culture work, which was performed to examine the direct effects of NE and DEX on ADRB2, demonstrated ADRB2 downregulation and MCP-1 upregulation in cultured EL-1 cells. Genotyping with regard to the ADRB2 genotype; rs1042714 CC vs CG/GG, on human saliva samples, showed that individuals with CC reported more anxiety following exposure to bullying behaviors as compared to the G carriers.

**Conclusion:**

We conclude that workplace bullying promotes anxiety and threaten well-being through an ADRB2 associated mechanism.

## Introduction

Exposure to bullying behaviors at the workplace by one’s peers or superiors has been recognized as a strong social stressor with a global prevalence of about 15% (Nielsen et al. 2010). The term “workplace bullying” refers to a systematic form of exposure to workplace mistreatment by other organization members where the target feels unable to defend him/herself (Einarsen and Skogstad 1996; Gredler 2003 Einarsen 2020). Bullying is not an either or phenomenon, but rather a gradually escalating and long-lasting process ranging from exposure to occasional acts of incivility to systematic and frequent exposure to aggression and social exclusion at work. Systematic reviews and meta-analyses of longitudinal studies have firmly documented that bullying is detrimental to the health and well-being of those targeted, for review see (Nielsen et al. 2014; Verkuil et al. 2015).

Specifically, the evidence demonstrates that exposure to workplace bullying is a significant risk factor for developing symptoms of depression, anxiety and insomnia (Nielsen and Einarsen 2012; Nielsen et al. 2014; Verkuil et al. 2015; Rajalingam et al. 2019). However, the understanding of how biological mechanisms are being affected and how adaptation transitions to mal-adaptation following exposure to social stressors such as workplace bullying, is limited. To attain a deeper understanding of the bullying phenomena, more attention and effort need to be directed towards investigating the underlying biological mechanisms, which involve both physiological and behavioral changes. The analyses presented here focus on examining the physiological changes that occur following exposure to social stressors, i.e., workplace bullying in humans and repeated social defeat in rats, and what impact such experiences may have for health and behavior. To also document casual mechanisms on a cellular level, an in vitro cell culture experiment was conducted.

There are strong theoretical reasons for why bullying mechanisms are important with regard to explaining the health outcomes of workplace bullying. Ongoing social stress such as exposure to workplace bullying in humans probably elicits activation of both the hypothalamus–pituitary–adrenal (HPA) axis and the sympathetic nervous system (SNS), for review see (Reader et al. 2015; Lowrance et al. 2016). This form of strong social stressors leads to peripheral release of norepinephrine (NE) and corticosteroids (CORTs) that induce complex immunological changes (Avitsur et al. 2001; Lowrance et al. 2016) such as increased release of monocyte chemoattractant protein-1 (MCP-1) from peripheral immune cells (Wood et al. 2015). Prolonged elevation of glucocorticoids (GCs) following chronic stress has been associated with brain region-dependent neuronal dysfunction, decreased synaptic density and impaired neuronal plasticity (Hall et al. 2015). In addition, the release of NE in the brain, from chromaffin cells in the adrenal medulla, and in lymphoid tissues that are innervated by efferent sympathetic nerve fibers (Felten et al. 1985), also involves physiological changes that may have an impact on behavior (Schmidt et al. 2007; Menard et al. 2017).

In particular, on-going threats from powerful individuals in the in-group, be it at work/school, in the clan or family may activate the stress responses described above. Therefore, repeated social defeat—which is an important component in bullying—most likely leads to a distinct release of NE. This results in NE activation of the adrenergic receptors, i.e., β_2_-adrenergic receptor (ADRB2) on target immune cells (Bierhaus et al. 2003). However, the effector functions depend on the nature of the stressor, its duration, and intensity. During chronic stress, ADRB2s expressed on target cell surfaces are exposed to NE for a prolonged time. Long-term NE exposure has been associated with desensitization of the receptor—a mechanism to reduce the cellular response (Hadcock and Malbon 1988). This results in a reduced number of receptors presented on the cell surface, affecting signaling pathways downstream of NE and ADRB2. In addition, genetic variation may play an important role for receptor functionality (Green et al. 1994; Naka et al. 2013).

Increasing evidence shows that genetic variation mediates individual differences in vulnerability and susceptibility to disease following exposure to stressors, including social defeat and exposure to ongoing workplace bullying (Aizawa et al. 2015; Santarelli et al. 2016; Jacobsen et al. 2019). Single nucleotide polymorphisms (SNPs), which refers to single nucleotide substitutions in the genetic material, definitely affect physiological responses to stressful stimuli. One important SNP, which may moderate the function of ADRB2, is the ADRB2 SNP rs1042714. This is a nonsynonymous SNP in the DNA located on the N-terminal tail (Glu27Gln) of the receptor that we know is associated with altered agonist binding, receptor activation, and agonist-promoted downregulation (Green et al. 1994). The observation that ADRB2 SNP rs1042714 directly affects the number of ADRB2 presented on the cell surface suggests that the ADRB2 genotype may affect the responses to stress, including bullying-induced negative health effects in humans.

Against this backdrop, we set out to investigate; (1) if or to what extent repeated social defeat may affect the circulating NE in animals and how this will affect further social behavior, (2) the direct effect of the stress hormone NE in cell culture and (3) how the SNP rs1042714 may moderate the effect of exposure to such on-going social stress as bullying constitutes in humans. We hypothesized that reduced ADRB2 signaling due to the ADRB2 genotype may predict the strength of the association between exposure to workplace bullying and anxiety.

## Methods

### Animals

A resident-intruder paradigm, where intruder Sprague Dawley rats were exposed to repeated social stress by dominant Long Evans resident rats for one hour each day for seven consecutive days, was used to study stress-induced changes in the HPA axis and the immune system. Ten male Long Evans rats (500–550 g) were housed with a female Long Evans rat (200–250 g) (Envigo; USA) in a 0.56 m^2^ cages. The ten male Sprague Dawley rats (300–400 g) used as intruders were housed in pairs, as were the ten male Sprague Dawley rats (300–400 g) used as controls (Janvier Labs; France). The different strains were kept in separate rooms. All rats were acclimatized to a 12:12 h light: dark cycle, ventilation rate of 15×  air per hour, 21–22 °C and 45–55% humidity. At all times, the rats had ad libitum access to food and water. Bedding was changed once a week. All animal procedures were approved by the Norwegian Food Safety Authority and performed in conformity with laws and regulations controlling experiments and procedures on live animals in Norway.

### Screening

To ensure dominant behavior of the Long Evans males, i.e., the resident rats in the paradigm, a screening against another group of Sprague Dawley rats was performed prior to the stress-conditioning week. The top ten aggressive rats were chosen based on the highest incidences of attacks over a period of 10 min.

### Resident-intruder paradigm

**Fig. 1:**
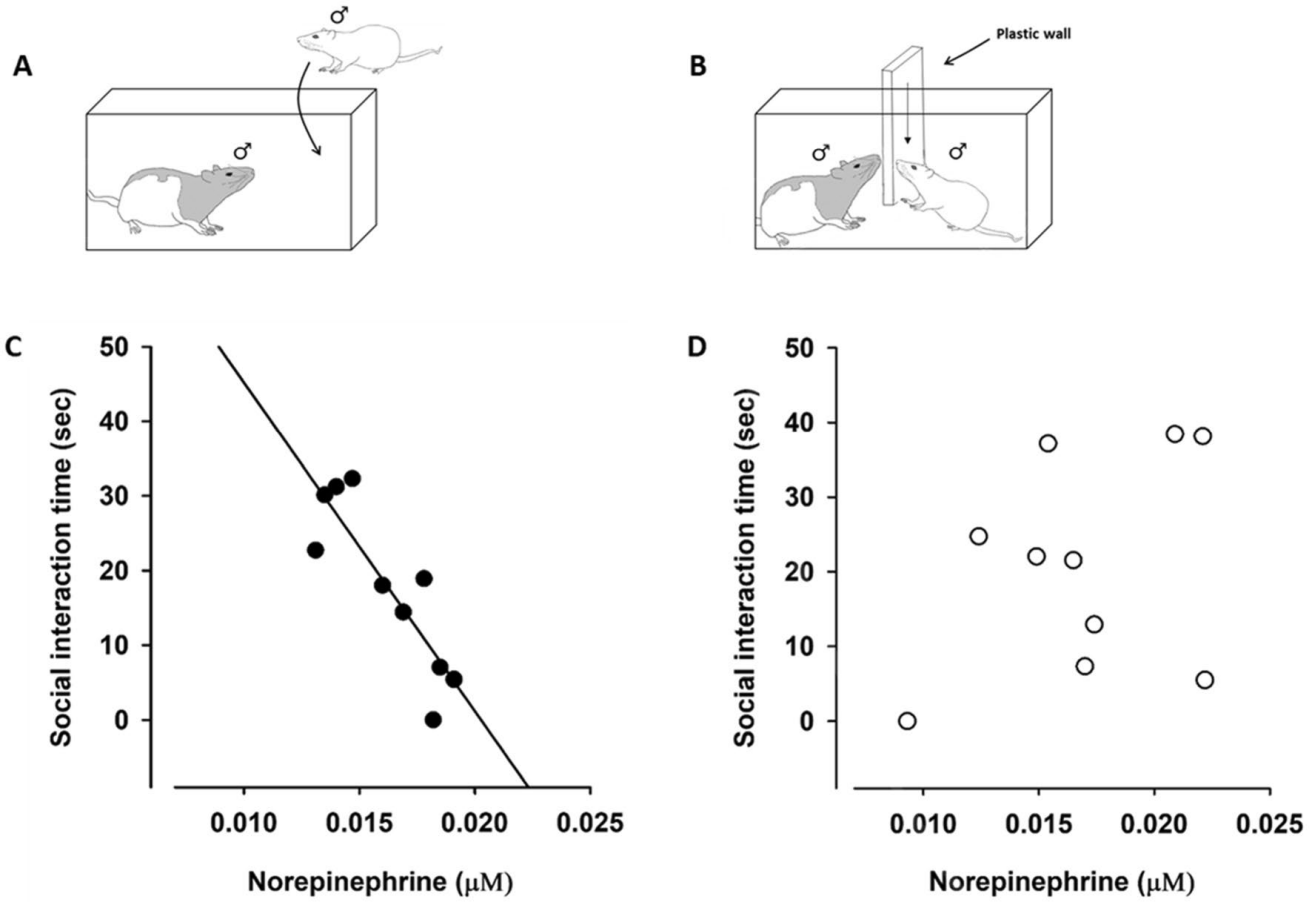
**A**, **B** As previously described, the resident intruder paradigm was used. **C** Linear regression analysis of correlation between norepinephrine concentrations (µM) in rat serum and social interaction time (sec) in stress exposed rats (black dots, linear regression; *R*^2^ = 0.741, *p* = 0.001) and in **D** control rats (white dots, linear regression; n.s)

As previously described (Rajalingam et al. 2020), the stress conditioning was performed by temporarily removing the female rat one hour prior to introducing the intruder animal into the resident cage. The rats were separated upon social defeat (submissive supine posture, freeze, or flight) or after 10 min of interaction by a perforated plastic wall (Fig. 1), allowing the intruder rat to still see, smell, and hear the resident rat. After 60 min in the resident cage, the intruder rat was returned to its home cage. The conditioning procedure described above was repeated for 7 days. To prevent habituation to the dominance establishment with the resident rat, the intruder animals were introduced to a new resident animal every day. The control animals followed the same procedure except that they visited an empty cage without a resident rat.

### Social interaction

A modified version of the social interaction test was used to assess the social interaction behavior of the Sprague Dawley rats (i.e., the test rats) following 1 week of stress or control conditioning (Kaidanovich-Beilin et al. 2011). The social interaction test was conducted 1 day after the last stress exposure, i.e., 24 h after the last episode of defeat. As previously described (Rajalingam et al. 2020), the test arena was a purpose made box (0.56 m^2^) divided into three compartments by two gated plastic walls and a small wire-like container in each flanking compartment. The test rats were allowed to habituate in the center compartment for 4 min before a novel rat of the same strain was placed into one of the small wire-like containers. The subsequent opening of the gates allowed the test rat to move freely between the compartments for 6 min. Movement and behavior of the test animals were recorded by a camera placed in a rack above the box. The social behavior, i.e., the time in direct social interaction (nose to nose) with the novel rat, was scored off-line after the experiments by a blinded technician.

### Anesthesia, blood and tissue harvesting

Following the social interaction test and one hour rest in their home cage, on day 8, the intruder Sprague Dawley and control rats were sedated with 5% isoflurane in air in a gas box prior to being moved to a 3% isoflurane anesthetic gas mask. The absence of withdrawal reflexes was considered sufficient anesthesia for surgery. The animal was fixated in a dorsal recumbence position and a v-cut through the skin and abdominal wall was made. A 10 mL syringe with a 1.2 mm cannula coated with 1.8 mg/mL EDTA (Sigma Life Science; Switzerland), was inserted into the left ventricle (cardiac puncture). Blood samples of 2 ml were drawn from the exposed and control Sprague Dawley rats. In accordance with the procedure previously described, 500 µL of the blood was immediately placed on liquid nitrogen for NE concentration measurements performed (Bergh et al. 2018). All Sprague Dawley rats were euthanized by dislocation of the neck under isoflurane anesthesia.

### Cultivation of in vitro cell line; EL-1 cells

The human macrophage cell line EL-1 was obtained from ATCC (Manassas, VA, USA) and maintained in Gibco™ IMDM medium with 4 mM L-glutamine adjusted to contain 1.5 g/L sodium bicarbonate supplemented with 0.05 mM 2-mercaptoethanol (Life Technologies, Grand Island, NY), 0.1 mM hypoxanthine (H-9377, Sigma-Aldrich), 0.016 mM thymidine (T-1895, Sigma-Aldrich), 10% fetal bovine serum (FBS) (Life Technologies, Grand Island, NY) and 1% Penicillin Streptomycin (Sigma-Aldrich Co. LLC, MO) in a humidified 5% CO_2_ atmosphere at 37 °C. The cell suspension was kept in T-75 TC flasks (Sarstedt, Nürnbrecht, Germany).

### Cell culture and treatment

EL-1 cells were seeded as suspensions of 0.5 × 10^6^ cells/well in 6-well plates (Sarstedt, Nürnbrecht, Germany) and exposed to 100 nM norepinephrine (ab120717, abcam, Cambridge, UK) for 24 h and/or 100 nM dexamethasone (D4902-25 MG, Sigma-Aldrich) for 3 h. For both exposures, no indication of cytotoxicity was observed (LDH toxicity assay: cytotoxicity < 0.2%, which is under the detection level). Norepinephrine was purchased as salt and solubilized in water to 100 mM, whereas dexamethasone was purchased as powder and dissolved in ethanol and cell culture medium (as recommended by the manufacturer) to obtain a stock concentration of 20 µg/mL. After 24 h, cells were separated from surrounding medium by centrifugation at 200×*g* for 5 min. The conditioned medium was collected and the cell pellet was directly lysed prior to storage at − 80 °C.

### RNA isolation and cDNA synthesis

RNA from the enriched myeloid cells and EL-1 cells was isolated according to the manufacturer’s protocol using the RNA/DNA purification kit (Norgen Biotek Cor.; Canada). Synthesis of cDNA from these tissues was carried out using the qScript cDNA synthesis kit (Quanta Biosciences Inc.; USA).

### Gene expression analyses

RNA quantification of the different genes was achieved by a two-step real time reverse transcription qPCR (RT-qPCR). Primer sequences (fwd,rev) were from Sigma Life Sciences, Switzerland: ADRB2 human (5′ CACTCCTCTTATTTGCTC3′ and 5′AAACTTTAGACTTTGC3′); β-actin (5′GACGACATGGAGAAATCTG3′ and 5′ATGATCTGGGTCATCTTCTC3′); PCR was run on Quantstudio 5 (Thermofisher Scientific; Norway) and analyzed using Quantstudio™ Design and Analysis Software.

### Luminex

Secretion of MCP-1 in conditioned medium from EL-1 cells was analyzed with Bio-Plex Pro™ cytokine assay (Bio-Rad Laboratories, Inc, Hercules, CA) on BioPlex MAGPIX (Luminex Corporation, Austin, TX) with Bio-Plex Manager™ MP Software (Bio-Rad Laboratories, Inc, Hercules, CA) according to the instruction manual. Briefly, this technique enables fast and accurate detection of cytokines in cell media by adding detection antibodies that will covalently couple to cytokines that are of interest. The streptavidin–phycoerythrin fluorescent reporter that is subsequently added binds to the detection antibody and provide a fluorescent light that can be quantified.

### Statistics–experimental part

The in vivo data of the rats were analyzed with linear regression (Sigmaplot 14.0), whereas the in vitro data from EL-1 cells were analyzed by linear mixed models (StataCorp. 2019. College Station, TX: StataCorp LLC). In all mixed models, treatment was included as a fixed effect, with control as a reference category. Furthermore, all mixed models included a random intercept for triplicates, to take into account the dependency between these observations.

### Collection of human data

A random sample of 5000 employees was drawn from The Norwegian Central Employee Register by Statistics Norway. The Norwegian Central Employee Register is the official register of all Norwegian employees, as reported by employers. Sampling criteria were adults from 18 to 60 years of age employed in a Norwegian enterprise. Questionnaires were distributed through the Norwegian Postal Service during spring 2015. Altogether 1608 persons returned the questionnaire (32%) and all respondents provided usable responses. Subjects who gave consent were also sent saliva collection kits. Among these, 1204 returned the saliva sample kit. The analyses were, however, performed with 1090 subjects due to missing data. The survey was approved by the Regional Committee for Medical Research Ethics for Eastern Norway. Responses were treated anonymously, and informed consent was given by the respondents.

Mean age was 45.19 (*SD* = 10.04) years with a range from 21 to 61 years. The sample consisted of slightly more women (52.1%) than men (47.8%). In total, 54.9% were married, 24.5% were common-law partners, 13.8% were unmarried, and 6.8% were widowed, separated, or divorced. Altogether 8.4% had less than 11 years of education, 30.8% had between 11 and 13 years, 32.3% had between 14 and 17 years, and 28.5% had 18 or more years. A total of 89.6% were in full-time employment, 6.6% were in part-time employment, 3.5% were on sick leave or occupational rehabilitation, and 0.3% were disabled pensioners or retired. Moreover, 36% had a leadership position with personnel responsibilities. Comparisons of sample characteristics with available data from Statistics Norway suggested that the sample distribution was somewhat skewed compared to the overall working population with regard to gender (53%men in population), educational level (less than 11 years of education: 17%; between 11 and 13 years: 42%; more than 14 years: 41% in the population), and age mean of 41.8 years in population. Hence, our sample is rather representative, yet somewhat on the older side and slightly less educated side, with some more females and fewer men.

### Instruments

Exposure to workplace bullying was measured with the 9-item short version of the *Negative Acts Questionnaire*—*Revised* (S-NAQ) inventory (Notelaers et al., 2019)*.* S-NAQ describes negative and unwanted behaviors that may be perceived as bullying if occurring on a regular basis. All items are formulated in behavioral terms and hence focus on the mere exposure to inappropriate behaviors while at work with no references to the term bullying. The S-NAQ contains items referring to both direct (e.g., openly attacking the victim) and indirect (e.g., social isolation, slander) behaviors (Einarsen et al. 2009). The items do also distinguish between personal- and work-related forms of bullying as well as acts of social exclusion (Einarsen et al. 2009). Example items are “Being ignored or excluded”, “Repeated reminders of your errors or mistakes”, and “Someone withholding information which affects your performance”. The respondents were asked to indicate how often they had been exposed to each specific item in questionnaire at their present worksite during the last 6 months. Response categories ranged from 1 to 5 (‘never’, ‘now and then’, ‘monthly’, ‘weekly’ and ‘daily’). This nine-item version of the S-NAQ had a Cronbach’s alpha of 0.86 in this study.

Five items from Hopkins Symptom Checklist (HSCL-25) reflecting typical symptoms of anxiety, i.e., “feeling fearful”, “nervousness or shakiness inside”, “heart pounding or racing”, “trembling” and “feeling tense or keyed up” during the last week were used. The HSCL is a valid and reliable (Rickels et al. 1976) self-administered instrument measuring mental distress (anxiety, depression, and psychosomatic complaints) in population surveys (Derogatis et al. 1974). Responses were given on a four-point scale, ranging from “1 = not at all” to “4 = extremely”. Cronbach’s alpha for this scale was 0.74 in the current study.

### Genotyping

As previously described (Jacobsen et al. 2018), genomic DNA was extracted from saliva using an OrageneRNA sample collection kit (DNA Genotech Inc. Kanata, Ontario, Canada). Single nucleotide polymorphism (SNP) genotyping was carried out using predesigned TaqMan SNP genotyping assays (Applied Biosystems, Foster City, CA, USA). Approximately 10 ng genomic DNA was amplified in a 5 µl reaction mixture in a 384-well plate containing 1 × TaqMan genotyping master mix (Applied Biosystems) and 1 × assay mix, the latter containing the respective primers and probes. The probes were labelled with the reporter dye FAM or VIC to distinguish between the two alleles. After initial denaturation and enzyme activation at 95 °C for 10 min, the reaction mixture was subjected to 40 cycles of 95 °C for 15 s and 60 °C for 1 min on an ABI 7900HT sequence detection system. Negative controls were included in every run. Genotypes were determined using the SDS 2.2 software (Applied Biosystems, Foster City, CA, USA). Approximately 10% of the samples were re-genotyped and the concordance rate was 100%.

### Statistical analysis–human cohort

Exposure to negative social acts was calculated using the mean-score of the 9-items in the S-NAQ inventory. In our sample we had 332 CC subjects, 539 CG subjects, and 219 GG subjects. Since we were interested in looking at the ADRB2 genotype mechanisms, the genotype was included as a dichotomous variable, CC versus CG/GG. To investigate the hypotheses about main and moderating effects, we conducted a moderation analysis using a modeling tool, SPSS; PROCESS v3.1, to test for linear associations between exposure to negative social acts and anxiety, as well as the interactive effects of negative social acts and ADRB2 genotype (CC versus CG/CC) with regard to anxiety. Deviation from the Hardy–Weinberg equilibrium was tested by the Chi-squared test.

As mentioned in the introduction, there are theoretical reasons for expecting that the impact and the magnitude of the relationship between workplace bullying on anxiety is conditioned by the ADRB2 rs1042714 genotype. Thus, the hypothesized moderation model was tested in full by means of the PROCESS macro (model 1) developed for SPSS. As previous research has established, age- (Christensen et al., 1999) and sex- (McLean et al., 2011) differences in anxiety were adjusted for in the analyses. A significant interaction term and a significant increase in explained variance (*R*^2^) were considered as indicative of an interaction effect.

As the scores on the S-NAQ (skewness: 4.18, kurtosis: 26.85) were non-normally distributed, all analyses were conducted using bootstrapping (5000 resamples). The bootstrap method has the advantage that it does not need to meet the assumptions of normality, equal variances, and homoscedasticity that are required in ordinary regression analyses. Multicollinearity was not an issue in the current study (VIF = 1.01). The level of significance was set to *p* < 0.05.

## Results

### Animals—stress-induced serum NE release

A clear association between the NE concentrations in rat serum and social interaction time in the stress-exposed group was demonstrated (Fig. 1C, black dots, linear regression; *R*^2^ = 0.741, *p* = 0.001). No such association was seen in the control group (Fig. 1D, white dots, linear regression; *R*^2^ = 0.125, *p* = 0.316).

### Cell culture experiments with EL-1 cells

**Fig. 2:**
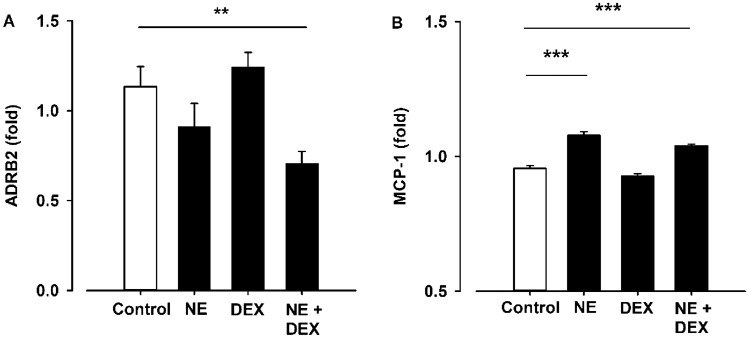
**A** Gene expression of the β_2_-adrenergic receptor (ADRB2) in EL-1 cells (human spleen resident myeloid cell line) exposed to norepinephrine (NE; 100 nM, 24 h) and dexamethasone (DEX; 100 nM, 3 h), *p *= 0.003. Gene expression data were normalized to β-actin. **B** Concentration measurements of monocyte chemoattractant protein-1 (MCP-1) in cell supernatants from EL-1 cells exposed to norepinephrine (100 nM, 24 h) and dexamethasone (100 nM, 3 h) measured by Luminex immunoassay, *p* = 0.001. **p < 0.01, ***p < 0.001, Linear mixed model

Gene expression analyses of EL-1 cells showed reduced expression of the ADRB2 following NE exposure. However, DEX exposure alone did not have any effect, the combination of NE and DEX treatment induced a significant downregulation of the ADRB2, compared to control conditions (Fig. 2A *p* = 0.003). Furthermore, only NE and NE + DEX exposure induced a significant increase of MCP-1 in the conditioned media from EL-1 cells (Fig. 2B *p* < 0.001).

### Humans—ADRB2 (rs1042714) genotype

The present data showed that 55% of the individuals included in our probability sample reported some exposure to negative acts; S-NAQ > 1 at the workplace during the last 6 months. Mean negative acts scores for men and women were; NAQ = 1.20 and 1.19, respectively. The mean anxiety scores for men were 1.31 and 1.36 for women.

The characteristics of the subjects are presented in Table 1. The genotyping analyses showed that 30.5% of the subjects had the ordinary variant CC, whereas the rest, i.e., 69.5% carried the rare variant CG/GG. No deviation from the Hardy–Weinberg equilibrium was observed (χ^2^ = 0.0001).

**Table 1:** | Characteristics of the subjects by ADRB2 genotype rs1042714; CC versus CG/GG

	Range	CC				CG/GG				Sum	*T* test
		N	%	Mean	SEM	N	%	Mean	SEM		
Subjects		332	30.5			758	69.5			1090	
Anxiety	1–4			1.36	0.021			1.34	0.014		0.80
NAQ	1–5			1.20	0.021			1.19	0.011		0.21
Age				45	0.559			45	0.366		
Male		167	50.0			358	49.4				
Female		163	50.0			385	51.9				
Education											
Secondary school or less		8	2.4			14	1.9				
High school		116	35.3			282	38.2				
University ≤ 4 years		111	33.7			236	31.9				
University ≥ 4 years		94	28.6			207	28.0				

**Fig. 3:**
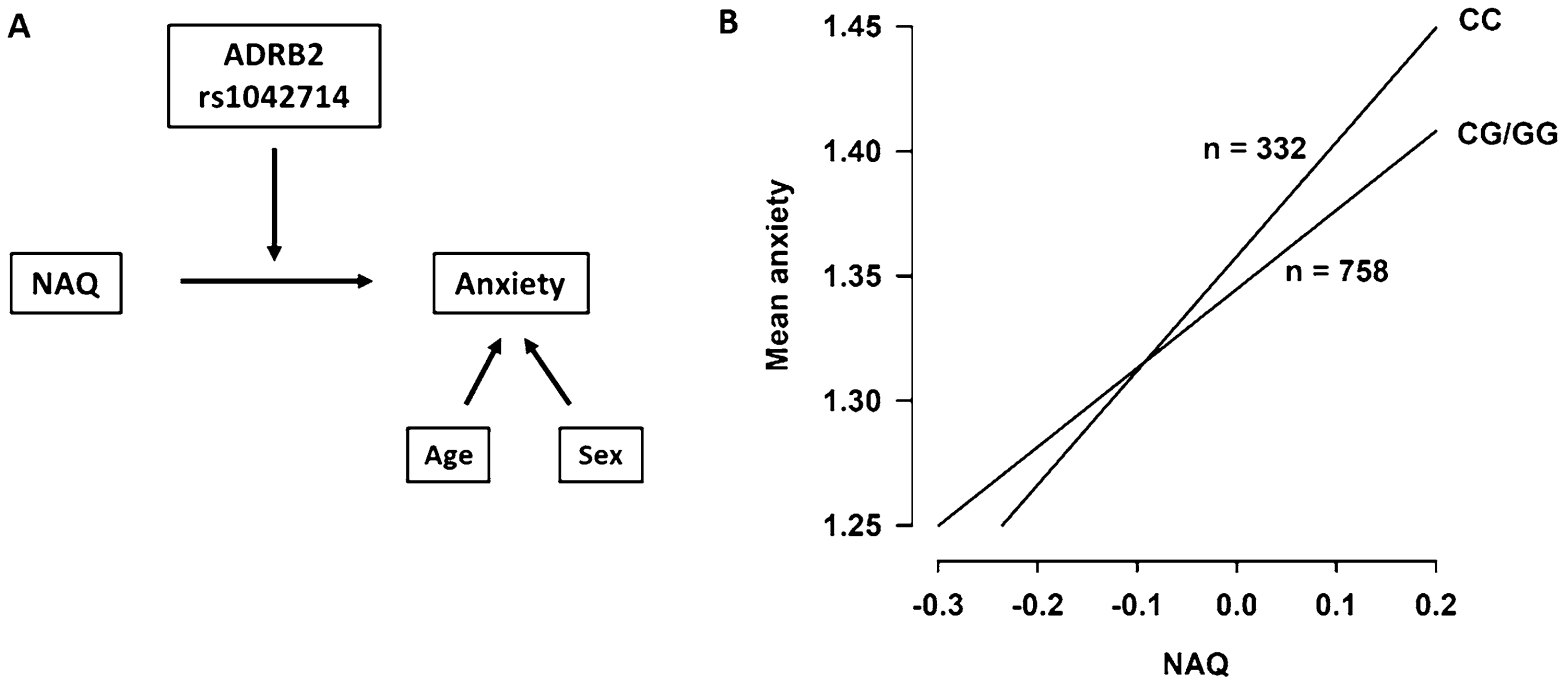
**A** A graphic illustration of the proposed relationship between workplace bullying and anxiety moderated by the β_2_-adrenergic receptor (ADRB2) genotype (adjusted for the covariates age and sex). **B** The relationship between negative social acts and mean anxiety after correction for age and sex. Subjects were divided into groups based on ADRB2 genotype rs1042714; CC versus CG/GG

Results from the moderation analysis is presented in Table 2. Analyses of the hypothesized model (Fig. 3A), i.e., associations between scores on NAQ and anxiety and the moderating effect of the ADRB2 genotype (CC versus CG/GG) on this relationship, showed that exposure to negative acts is significantly associated with anxiety (*B* = 0.36, *p* < 0.001). Furthermore, the significant interaction term NAQ*ADRB2 genotype (CC versus CG/GG) showed that the relationship between exposure to negative acts and anxiety was strengthened among carriers of the C allele.

**Table 2:** Regression analysis SPSS PROCESS model 1 with ADRB2 genotype rs1042714; CC versus CG/GG (bootstrapping with 5000 samples)

	B	SE	*p* value	95% CI	
Anxiety					
NAQ	0.3601	0.0350	0.000	0.2915	0.4287
ADRB2 CC* vs. CG/GG	− 0.0131	0.0247	0.5949	− 0.0615	0.0353
NAQ × ADRB2 CC* vs. CG/GG	− 0.1412	0.0698	0.0435	− 0.2782	− 0.0041
Age	− 0.0024	0.0011	0.0349	− 0.0046	− 0.0002
Sex	0.0683	0.0228	0.0028	0.0235	0.1132

The analysis were adjusted for the covariates age and sex

*B* beta coefficient, *SE*, standard error, *CI* confidence interval

*reference group

The present data suggest that the relationship between exposure to negative acts and anxiety is moderated by the ADRB2 genotype (CC versus CG/GG) and thereby strengthens the relationship among C allele carriers compared to individuals having the G allele (Fig. 3B). The model including the interaction term explained 12.3% of the variance in anxiety.

## Discussion

The present study showed a clear association between reduced social interaction and increased level of circulating NE in rats previously exposed to repeated social defeat. Taken together with our recent observation that repeated social defeat induced a persistent decrease in the myeloid ADRB2 gene expression of the same stress-exposed animals (Rajalingam et al. 2020), this suggests that exposure to ongoing and repeated social defeat may affect future social behavior in animals. Hence, our present in vivo and in vitro data point to a link between social stress, fear, social withdrawal, and NE-ADRB2 signaling. Moreover, our cell culture work on myeloid immune cells emphasized the role of MCP-1 as a downstream effect of ADRB2 activation. Given that the response to social stressors increases levels of circulating NE activating the ADRB2 receptors, this suggests that social stress also could facilitate MCP-1 driven immunological processes. The in vivo and in vitro arm of the present study, suggested that the function of NE-ADRB2 signaling and its possible link to the immune system needs to be taken into account to understand how workplace bullying may affect victims of workplace bullying. Thus, the present study also included a human arm addressing the role of the ADRB2. For the first time, we show that the association between exposure to workplace bullying and anxiety was moderated by the human ADRB2 SNP rs1042714 C > G. Interestingly, carriers of two C alleles, supposed to have decreased membrane-bound ADRB2, reported more anxiety than did the G allele carriers following exposure to acts of workplace bullying. Hence, the present work shows that persistent social stressors such as workplace bullying may be linked to a NE-ADRB2 associated mechanism.

Regarding the link to the immune system previous observations show that the communication between the central nervous system (CNS) and the immune cells is bidirectional (Niraula et al. 2018; Yin et al. 2019), for review see (Lorton et al. 2006; Reader et al. 2015). Stress-induced NE and CORT stimulate immune cells and trigger the activation of inflammatory processes. Whereas CORT mediates negative feedback loop at all levels of the HPA axis and suppresses inflammatory processes in activated immune cells, NE activates anti-inflammatory processes through ADRB2 in immune cells (Farmer and Pugin 2000; McNamee et al. 2010). Under normal circumstances, these regulatory mechanisms function to reinstate homeostasis. However, during chronic stress, regulatory mechanisms may become mal-adaptive. Thus, long-term stress has been associated with negative health effects such as low-grade systemic inflammation, insomnia, depression, and anxiety (Brousse et al. 2008; Menard et al. 2017; Jacobsen et al. 2019; Rajalingam et al. 2019).

Studies on chronic stress in murine models have demonstrated elevated production of pro-inflammatory cytokines (Voorhees et al. 2013; Niraula et al. 2018). Increased production of leukocytes in the bone marrow (BM) and egress of immune cells from the BM to peripheral lymphoid tissues, i.e., the spleen, has also been demonstrated in stress-induced rats (Engler et al. 2004; Yin et al. 2019). Examination of circulating immune cells from these animals have revealed glucocorticoid resistance and increased production of pro-inflammatory cytokines (Bailey et al. 2004; Miller et al. 2008). These immune cells have been shown to be recruited to the blood–brain-barrier (BBB) where cytokines are being released, in which CNS resident immune cells, i.e., microglia, may become affected (Wohleb et al. 2014), for review see (Wohleb et al. 2015). It is believed that peripheral cytokines passing the BBB affect specific neuro-circuits, thus leading to pathophysiology and psychiatric illnesses such as depression and anxiety (Reader et al. 2015; Réus et al. 2015). The present study adds to this by demonstrating that also MCP-1 driven downstream processes might be involved following stress-induced ADRB2 activation.

NE signaling through the ADRB2 has been associated with immune suppression (Farmer and Pugin 2000; Hanke et al. 2012). However, long-term NE exposure causing receptor desensitization may diminish the suppressive effects (Hadcock and Malbon 1988). As previously shown, a reduced ADRB2 gene expression in stress-induced rats compared with control rats was demonstrated (Rajalingam et al. 2020). Similarly, the NE and NE + DEX treated EL-1 cells—but not DEX treatment alone—showed reduced ADRB2 expression supporting the hypothesis that persistent NE exposure affects receptor responsivity. In addition, the observation of increased levels of MCP-1 in the conditioned media, from NE and/or DEX-treated EL-1 cells, underpin the idea that the immune suppression is attenuated by persistent NE exposure. The ADRB2 SNP rs1042714 had a significant moderating effect on the association between exposure to bullying and anxiety in the sample of workers. This links the NE-ADRB2 system to the mechanisms. Interestingly, individuals with two C alleles, which may have stronger agonist-driven desensitization, showed a strengthened relationship between exposure to workplace bullying and anxiety than G allele carriers. Taken together, this clearly point to mechanisms where stress, through activation of the HPA axis and the immune system, may affect physical health.

Although more research is needed, and the data presented in the present study should be interpreted by caution, our observations suggest that the G allele carriers are more robust, i.e., more resilient against developing anxiety, than individuals with two C alleles. Hence, our findings fit well with the earlier observation that the rs1042714 SNP G allele prevents desensitization following long-term agonist exposure (Green et al. 1994). As such, it is tempting to speculate that less desensitization observed in G carriers—which we here consider health-promoting following persistent stressor exposure—contribute to the maintenance of the NE driven immunosuppressive processes. The present study, therefore, suggests that exposure to social stressors that in our animal model elicit fear, that also, based on the present cell culture work, seems to facilitate NE/MCP-1-driven immunological processes, may promote depression and anxiety.

In summary, our study supports the theory that strong social stressors such as workplace bullying may involve activation of the neuro-immune interface and have severe physiological consequences. Interestingly, the present data suggest that exposure to social stressors may trigger the immune system through ADRB2 and also links the low membrane-bound ADRB2 rs1042714 C allele genotype to stress-induced anxiety in humans. We conclude that workplace bullying promotes anxiety and threatens well-being through an NE-ADRB2 associated mechanism.

## Data Availability

The datasets supporting the conclusions of this article are included within the article and its additional files.
